# Alteration of circulating natural autoantibodies to CD25-derived peptide antigens and FOXP3 in non-small cell lung cancer

**DOI:** 10.1038/s41598-018-28277-1

**Published:** 2018-06-29

**Authors:** Huan Zhao, Xuan Zhang, Zhifeng Han, Wenjing Xie, Wei Yang, Jun Wei

**Affiliations:** 1 Second Hospital of Jilin University, Changchun, 130041 China; 2 Department of Thoracic Surgery, China-Japan Union Hospital, Jilin University, Changchun, 130031 China; 3 Institute of Health Research & Innovation, University of the Highlands & Islands, Centre for Health Science, Inverness, IV2 3JH UK

## Abstract

Natural autoantibody is a key component for immune surveillance function. Regulatory T (Treg) cells play indispensable roles in promoting tumorigenesis via immune escape mechanisms. Both CD25 and FOXP3 are specific markers for Treg cells and their natural autoantibodies may be involved in anticancer activities. This work was designed to develop an in-house enzyme-linked immunosorbent assay (ELISA) to examine plasma natural IgG against CD25 and FOXP3 in non-small cell lung cancer (NSCLC). Compared with control subjects, NSCLC patients had significantly higher levels of plasma IgG for CD25a (Z = −8.05, *P* < 0.001) and FOXP3 (Z = −4.17, *P* < 0.001), lower levels for CD25b (Z = −3.58, *P* < 0.001), and a trend toward lower levels for CD25c (Z = −1.70, *P* = 0.09). Interestingly, the anti-CD25b IgG assay had a sensitivity of 25.0% against a specificity of 95.0% in an early stage patients (T_1_N_0_M_0_) who showed the lowest anti-CD25b IgG levels among 4 subgroups classified based on staging information. Kaplan-Meier survival analysis showed that patients with high anti-FOXP3 IgG levels had shorter survival than those with low anti-FOXP3 IgG levels (χ^2^ = 3.75, *P* = 0.05). In conclusion, anti-CD25b IgG may be a promising biomarker in terms of screening individuals at high risk of lung cancer.

## Introduction

Epidemiological study reported that there were about 14.1 million newly-diagnosed cancer cases and 8.2 million cancer-related deaths in 2012^1^. Of all types of malignant tumors, lung cancer is the most frequently diagnosed malignancy and the most common cause of cancer related-mortality worldwide, accounting for nearly 25% of all cancer deaths^1^. Lung cancer is also the leading cause of all cancer deaths in China^2^ and has been classified into several subtypes, in which non-small cell lung cancer (NSCLC) accounts for approximately 85% of all lung cancer cases^3^. While the development of new treatment regimens played an active role in the treatment of lung cancer patients, the outcomes reported to date have not been satisfactory and the 5-year survival rates remain less than 15%^4^. When lung cancer was identified in an early stage, the 5-year survival rate could achieve up to 50%^5^. Therefore, there is an urgent need to develop a screening test among individuals at high risk of lung cancer.

Regulatory T (Treg) cells, naturally produced in part by the thymus as a subpopulation of functional mature T cells, play an indispensable role in maintaining immune tolerance and regulating immune response^6^. CD25 also known as the interleukin (IL)-2 receptor α-chain, is a phenotypic marker of CD4^+^ Treg cells^7^. Forkhead box P3 (FOXP3), a transcriptional factor, is another marker of Treg cells, which may be associated with developing suppressive function of Treg cells^8^. Deficiency or functional deficit of CD4^+^CD25^+^ Treg cells can cause the development of various autoimmune diseases^9^. CD4^+^CD25^+^ Treg cells are also involved in promoting the evasion of cancer cells from immune surveillance. So they can impede anti-tumor response through suppression of the activation, expansion and proliferation of tumor-specific effector T cells including cytotoxic CD8^+^T cell^10^, CD4^+^T cell and NK cell^11,12^. Depletion of Treg cells before tumor challenge resulted in tumor regression^13,14^. High infiltration of Treg cells in tumor microenvironment has been found to be associated with poor outcomes in multiple solid tumors^15^.

Natural autoantibodies are mainly produced by B-1 cells in the absence of external stimulation^16^, and have a series of physiological activities such as maintaining homeostasis of the immune system^17^, pathogen elimination^18^, regulating B cell development^19^ and immune defense^20^. Because of an age-related reduction of natural autoantibodies, a decline of its ability to remove noxious molecules might be associated with the development and progression of several chronic illnesses commonly occurring in middle-age or older people such as neurodegeneration, type-2 diabetes, atherosclerosis and malignant diseases^21^. Therefore, researchers have suggested that natural autoantibodies could be used as biomarkers for identification of immune-related conditions such as cancer^17^.

Circulating natural autoantibodies for CD25-derived peptide antigens have been reported to be elevated in several types of cancer including breast cancer^22^, esophageal cancer^23^ and lung cancer^24,25^. Meanwhile, several studies have reported an increase in anti-FOXP3 IgG levels and FOXP3 mRNA expression in different types of cancer^26,27^. However, the mechanism behind increased anti-CD25 and anti-FOXP3 antibody levels in cancer patients have not be fully addressed. The present study was thus undertaken to detect circulating levels of natural autoantibodies against peptide antigens derived from CD25 and FOXP3 with an enzyme-linked immunosorbent assay (ELISA) developed in-house and to confirm if circulating anti-CD25 and anti-FOXP3 autoantibodies have diagnostic values for early detection of NSCLC.

## Results

The in-house ELISA showed a good reproducibility with coefficients of variation (CV) of 11.9% from anti-CD25a IgG assay, 13.9% from anti-CD25b IgG assay, 13.9% from anti-CD25c IgG assay, and 12.3% from anti-FOXP3 IgG assay (Supplementary Table S1).

### Changes of plasma anti-CD25 and anti-FOXP3 IgG levels in NSCLC

As shown in Table 1, plasma anti-CD25a IgG levels were significantly higher in patients with NSCLC than control subjects (Z = −8.05, *P* < 0.001), both male and female patients contributing to the increased anti-CD25a I gG levels (Z = −7.90, *P* < 0.001 in males and Z = −2.89, *P* = 0.004 in females, respectively). There was a decrease in anti-CD25b IgG levels in NSCLC patients compared with control subjects (Z = −3.58, *P* < 0.001), especially in female patients (Z = −4.72, *P* < 0.001). Plasma anti-CD25c IgG levels were lower in NSCLC patients than control subjects but failed to show statistically significant (Z = −1.70, *P* = 0.09). Plasma anti-FOXP3 IgG levels were significantly higher in the patient group than the control group (Z = −4.17, *P* < 0.001), in which male patients mainly contributed to the increased anti-FOXP3 IgG levels (Z = −4.14, *P* < 0.001). When these subjects were divided into two subgroups based on their ages, one ≥60 years old subgroup and the other one <60 years old subgroup, decreased anti-CD25b IgG levels were shown only in patients aged < 60 years (Z = −3.92, *P* < 0.001), while increased anti-FOXP3 IgG levels were found mainly in patients aged ≥60 years (Table 2). Although both major types of NSCLC contributed to an increase in plasma anti-CD25a and anti-FOXP3 IgG levels (Table 3), decreased anti-CD25b IgG levels were observed only in patients with adenocarcinoma (Z = −4.22, *P* < 0.001).

**Table 1:** The levels of plasma IgG against CD25 and FOXP3 in patients with NSCLC and control subjects.

IgG	Group	Patient (n)	Control (n)	Z^a^	*P* ^b^
CD25a	Male	0.69 ± 0.17 (131)	0.50 ± 0.16 (103)	−7.90	<0.001
	Female	0.61 ± 0.18 (80)	0.54 ± 0.17 (97)	−2.89	0.004
	Both	0.66 ± 0.18 (211)	0.52 ± 0.16 (200)	−8.05	<0.001
CD25b	Male	0.41 ± 0.24 (131)	0.41 ± 0.20 (103)	−0.66	0.51
	Female	0.32 ± 0.16 (80)	0.46 ± 0.23 (97)	−4.72	<0.001
	Both	0.37 ± 0.22 (211)	0.43 ± 0.22 (200)	−3.58	<0.001
CD25c	Male	1.09 ± 0.31 (131)	1.11 ± 0.29 (103)	−0.69	0.49
	Female	1.07 ± 0.32 (80)	1.17 ± 0.34 (97)	−1.73	0.08
	Both	1.08 ± 0.31 (211)	1.14 ± 0.32 (200)	−1.70	0.09
FOXP3	Male	0.63 ± 0.24 (131)	0.51 ± 0.23 (103)	−4.14	<0.001
	Female	0.56 ± 0.21 (80)	0.53 ± 0.26 (97)	−1.42	0.16
	Both	0.60 ± 0.23 (211)	0.52 ± 0.24 (200)	−4.17	<0.001

Plasma IgG levels are expressed as mean ± SD in SBR.

^a^Mann–Whitney *U* test (two-tailed). ^b^*P* < 0.0125 was considered to be statistically significant as four individual antigens were tested.

**Table 2:** The levels of plasma IgG against CD25 and FOXP3 in different age subgroups.

IgG	age (years)	Patient (n)	Control (n)	Z^a^	*P* ^b^
CD25a	≥60	0.68 ± 0.17(106)	0.52 ± 0.17 (99)	−6.06	<0.001
	<60	0.65 ± 0.18(105)	0.52 ± 0.16 (101)	−5.21	<0.001
CD25b	≥60	0.40 ± 0.23(106)	0.42 ± 0.21 (99)	−1.16	0.25
	<60	0.35 ± 0.19(105)	0.45 ± 0.23 (101)	−3.92	<0.001
CD25c	≥60	1.12 ± 0.33(106)	1.14 ± 0.30 (99)	−0.64	0.52
	<60	1.05 ± 0.29(105)	1.13 ± 0.34 (101)	−1.73	0.08
FOXP3	≥60	0.62 ± 0.23(106)	0.53 ± 0.26 (99)	−3.53	<0.001
	<60	0.58 ± 0.23(105)	0.52 ± 0.23 (101)	−2.32	0.02

Plasma IgG levels are expressed as mean ± SD in SBR.

^a^Mann–Whitney *U* test (two-tailed). ^b^*P* < 0.0125 was considered to be statistically significant as four individual antigens were tested.

**Table 3:** The levels of plasma IgG antibodies against CD25 and FOXP3 in two histological types of NSCLC.

IgG	Patient (n)	Control (n)	Z^a^	*P* ^b^
**CD25a**				
Squamous	0.69 ± 0.17(87)	0.52 ± 0.16 (200)	−7.55	<0.001
Adenocarcinoma	0.64 ± 0.18(124)	0.52 ± 0.16 (200)	−5.88	<0.001
**CD25b**				
Squamous	0.40 ± 0.21(87)	0.43 ± 0.22(200)	−1.31	0.19
Adenocarcinoma	0.36 ± 0.22(124)	0.43 ± 0.22(200)	−4.22	<0.001
**CD25c**				
Squamous	1.11 ± 0.34(87)	1.14 ± 0.32(200)	−0.70	0.49
Adenocarcinoma	1.06 ± 0.29(124)	1.14 ± 0.32(200)	−1.95	0.05
**FOXP3**				
Squamous	0.62 ± 0.21(87)	0.52 ± 0.24(200)	−4.32	<0.001
Adenocarcinoma	0.59 ± 0.25(124)	0.52 ± 0.24(200)	−2.71	0.007

Plasma IgG levels are expressed as mean ± SD in SBR.

^a^Mann–Whitney *U* test (two-tailed); ^b^*P* < 0.0125 was considered to be statistically significant as four individual antigens were tested.

Further analysis was performed to explore the differences in plasma IgG levels for CD25 and FOXP3 between four subgroups classified based on staging information. As shown in Table 4, both anti-CD25a and anti-FOXP3 IgG levels were significantly elevated in groups II, III and IV rather than group I, when compared with the control group; however, anti-CD25b IgG levels were found to be significantly decreased in groups I and II instead of groups III and IV. The total IgG level in NSCLC patients did not show significantly different from that in control subjects (3.00 ± 1.14 mg/ml in the patient group and 3.10 ± 1.08 mg/ml in the control group, Z = −0.73, *P* = 0.46).

**Table 4:** The levels of circulating antibodies against CD25 and FOXP3 in four groups of NSCLC.

TAAs	Group^a^	Patient (n)	Control (n)	Z^b^	*P* ^*c*^
CD25a	I	0.61 ± 0.22(20)	0.52 ± 0.16(200)	−1.79	0.07
	II	0.65 ± 0.17(101)	0.52 ± 0.16(200)	−6.32	<0.001
	III	0.70 ± 0.15(41)	0.52 ± 0.16(200)	−5.97	<0.001
	IV	0.68 ± 0.19(49)	0.52 ± 0.16(200)	−5.05	<0.001
CD25b	I	0.31 ± 0.16(20)	0.43 ± 0.22(200)	−2.63	0.009
	II	0.37 ± 0.22(101)	0.43 ± 0.22(200)	−3.31	0.001
	III	0.35 ± 0.14(41)	0.43 ± 0.22(200)	−2.13	0.03
	IV	0.43 ± 0.26(49)	0.43 ± 0.22(200)	−0.82	0.41
CD25c	I	1.14 ± 0.37(20)	1.14 ± 0.32 (200)	−0.32	0.75
	II	1.05 ± 0.28(101)	1.14 ± 0.32 (200)	−2.12	0.03
	III	1.11 ± 0.30(41)	1.14 ± 0.32 (200)	−0.05	0.96
	IV	1.10 ± 0.35(49)	1.14 ± 0.32 (200)	−0.95	0.34
FOXP3	I	0.55 ± 0.28(20)	0.52 ± 0.24(200)	−0.38	0.71
	II	0.60 ± 0.25(101)	0.52 ± 0.24(200)	−3.19	0.001
	III	0.60 ± 0.18(41)	0.52 ± 0.24(200)	−2.98	0.003
	IV	0.62 ± 0.22(49)	0.52 ± 0.24(200)	−3.15	0.002

Plasma IgG levels are expressed as mean ± SD in SBR.

^a^Group I for stage T_1_N_0_M_0_, group II for stage T_1_N_1_M_0_ + T_2_N_0_M_0_, group III for stage T_2_N_1_M_0_ + T_3_N_0_M_0_ and group IV for stages 3 and 4; ^b^Mann–Whitney *U* test (two-tailed); ^c^*P* < 0.0125 was considered to be statistically significant as three individual antigens were tested.

**Figure 1:**
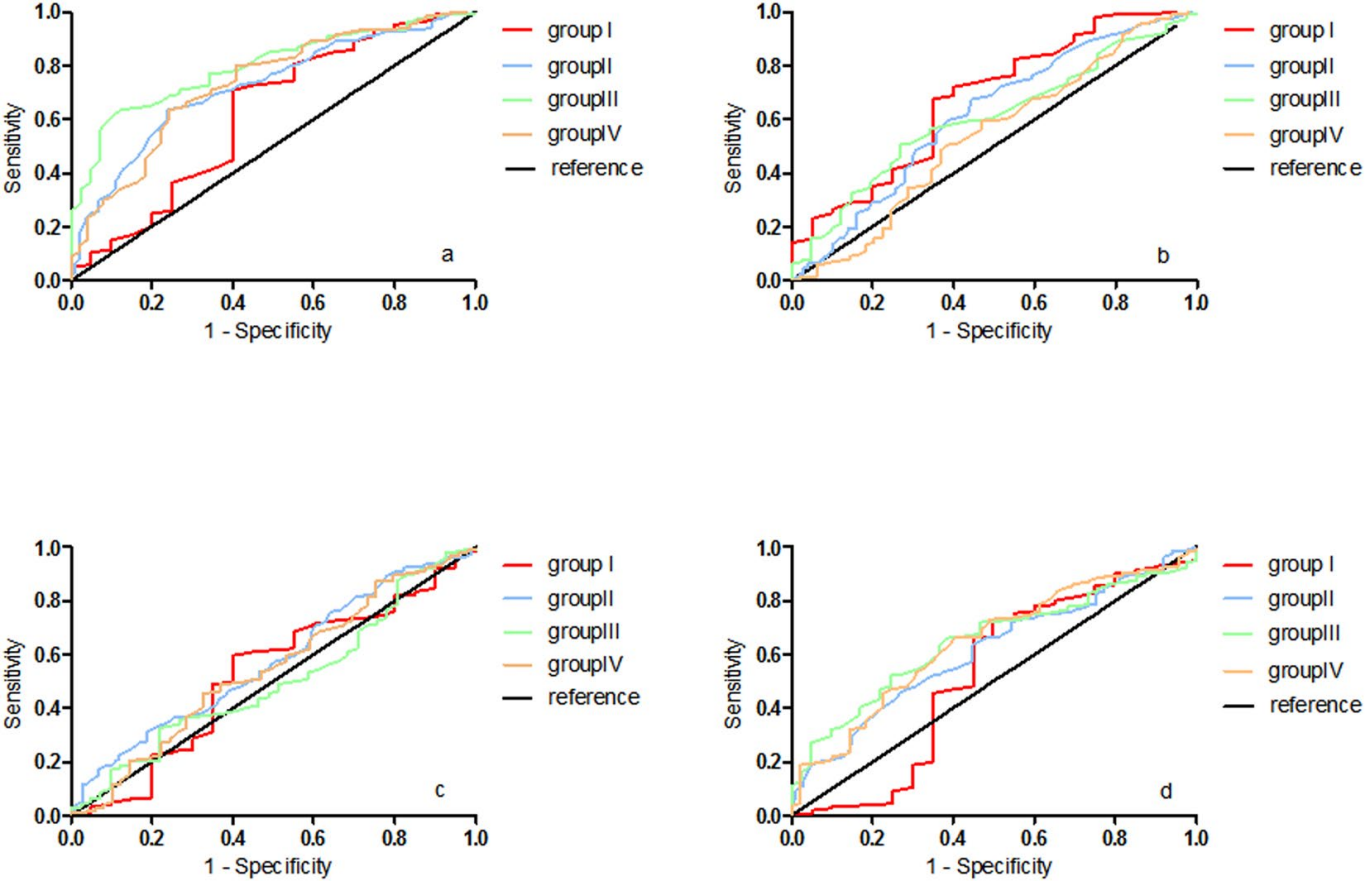
ROC curve analysis of circulating anti-CD25 and anti-Foxp3 IgG levels in different subgroups of NSCLC. Plasma anti-CD25a IgG levels; (**b**) Plasma anti-CD25b IgG levels; (**c**) Plasma anti-CD25c IgG levels; (**d**) Plasma anti-FOXP3 IgG levels.

Receiver operating characteristic (ROC) curve analysis showed an area under the ROC curve (AUC) of 0.73(95% CI 0.68–0.78), with sensitivity of 14.2 against a specificity of 95.0% for anti-CD25a IgG assay, an AUC of 0.60 (95% CI 0.55–0.66), with sensitivity of 14.2 for anti-CD25b IgG assay, an AUC of 0.55(95% CI 0.49–0.60), with sensitivity of 8.1 for anti-CD25c IgG assay,and an AUC of 0.62(95% CI 0.57–0.67), with sensitivity of 5.7 for anti-FOXP3 IgG assay (Table 5 and Fig. 1).

**Table 5:** ROC analysis of circulating antibodies against CD25 and FOXP3 in four subgroups of NSCLC.

TAAs	Group	AUC	SE^a^	95%CI	Sensitivity (%)^b^
CD25a	I	0.62	0.075	0.47–0.77	20.0
	II	0.72	0.03	0.66–0.78	10.9
	III	0.80	0.033	0.73–0.86	17.1
	IV	0.73	0.04	0.65–0.81	16.3
	Overall	0.73	0.025	0.68–0.78	14.2
CD25b	I	0.68	0.067	0.55–0.81	25.0
	II	0.62	0.035	0.55–0.69	14.9
	III	0.61	0.045	0.52–0.70	7.3
	IV	0.54	0.049	0.44–0.63	14.3
	Overall	0.60	0.028	0.55–0.66	14.2
CD25c	I	0.52	0.072	0.38–0.66	5.0
	II	0.58	0.035	0.51–0.64	8.9
	III	0.50	0.049	0.41–0.60	7.3
	IV	0.54	0.047	0.45–0.64	8.2
	Overall	0.55	0.028	0.49–0.60	8.1
FOXP3	I	0.53	0.08	0.36–0.69	5.0
	II	0.61	0.03	0.55–0.68	7.9
	III	0.65	0.04	0.57–0.73	2.4
	IV	0.65	0.04	0.56–0.73	4.1
	Overall	0.62	0.03	0.57–0.67	5.7

^a^Standard error; ^b^against a specificity of 95.0%.

Interestingly, plasma anti-CD25b IgG assay showed the sensitivity of 25.0% against a specificity of 95.0% in group I (T_1_N_0_M_0_).

### The relationship between overall survival and plasma IgG levels

**Figure 2:**
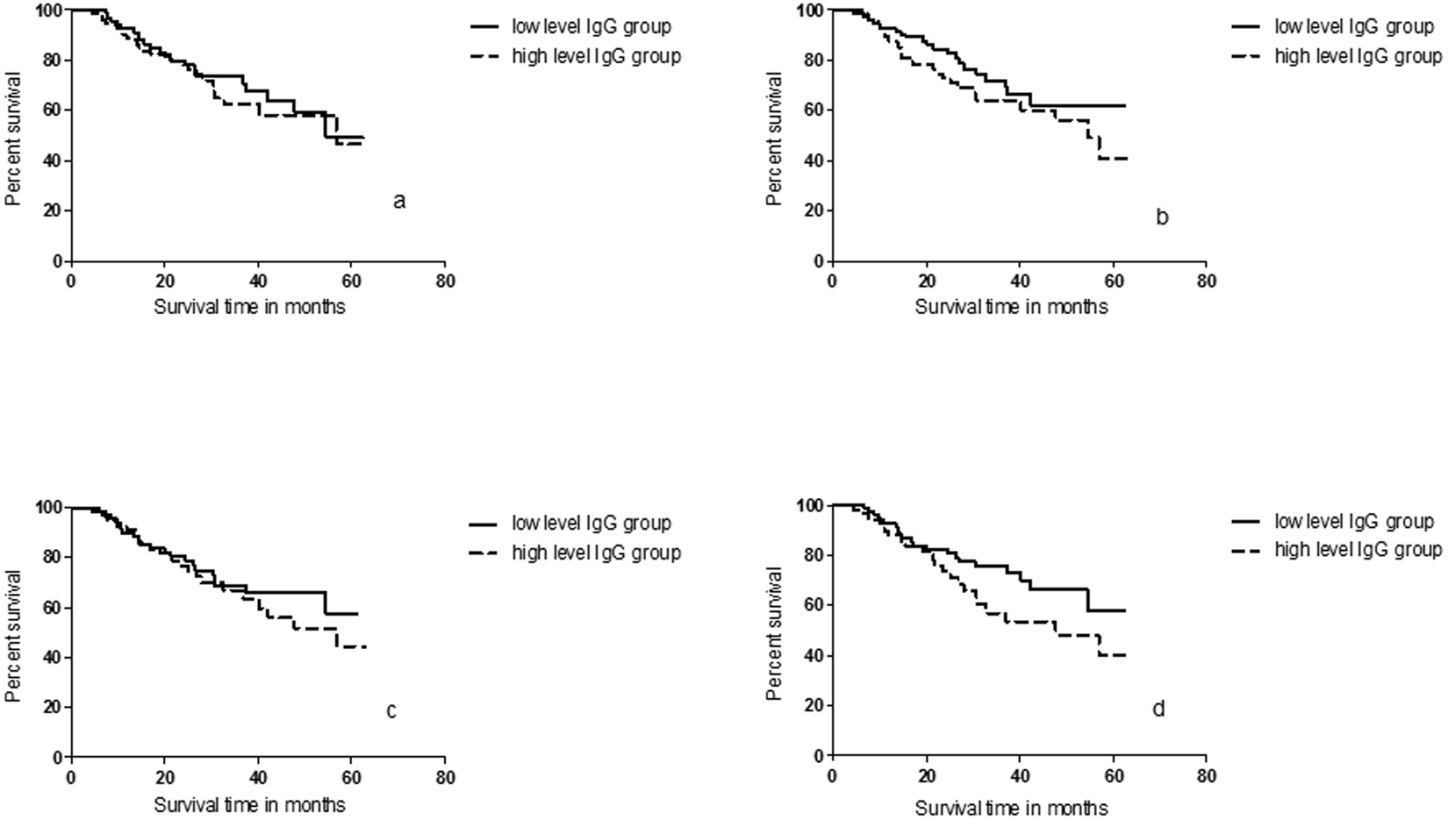
Kaplan-Meier survival analysis of difference in overall survival between NSCLC patients with high IgG levels and those with low IgG levels. (**a**) Plasma anti-CD25a IgG levels. (**b**) Plasma anti-CD25b IgG levels. (**c**) Plasma anti-CD25c IgG levels; (**d**) Plasma anti-FOXP3 IgG levels.

By the end of 2017, 154 of 211 NSCLC patients were successfully followed up with confirmation of 52 deaths. Kaplan-Meier survival analysis showed no difference in mean survival between NSCLC patients with high anti-CD25 IgG levels and those with low anti-CD25 IgG levels (χ^2^ = 0.09, *P* = 0.76 for anti-CD25a IgG, χ^2^ = 0.79, *P* = 0.38 for anti-CD25b IgG and χ^2^ = 0.38, *P* = 0.54 for anti-CD25c IgG). However, NSCLC patients with high anti-FOXP3 IgG levels had shorter survival than those with low anti-Foxp3 IgG levels (χ^2^ = 3.75, P = 0.05), but the statistical significance failed to survive the correction for age, sex, stages and histological types of NSCLC (Table 6 and Fig. 2). Analysis with univariate cox proportional hazard model showed that NSCLC subgroup was the only factor significantly correlated with overall survival (Table 7).

**Table 6:** Kaplan-Meier survival analysis of differences in overall survival (OS) between patients with low IgG levels and those with high IgG levels.

OS (months)^a^				
IgG	Low-level group	High-level group	χ^2 b^	*P* ^c^
CD25a	46.6 ± 2.74	45.1 ± 2.78	0.09	0.76
CD25b	48.2 ± 2.63	44.0 ± 2.81	0.79	0.38
CD25c	46.7 ± 2.61	44.7 ± 2.79	0.38	0.54
FOXP3	48.5 ± 2.46	42.4 ± 3.06	3.75	0.05

^a^Mean ± SE in OS; ^b^Calculated from Cox regression analysis; ^c^Uncorrected *P*-value for age, gender, NSCLC stages and types.

**Table 7:** Univariate cox proportional hazard model for overall survival.

Variable	HR	95%CI	*P*
Age (≥ 60 yr vs <60 yr)	1.23	0.69–2.18	0.48
Gender (male vs female)	0.92	0.51–1.68	0.79
Subgroup (I–III vs IV)	2.41	1.33–4.38	0.004
Histology (Squamous vs adenocarcinoma)	0.93	0.52–1.68	0.81
Anti-CD25a (low-level group vs high-level group)	0.86	0.48–1.53	0.61
Anti-CD25b (low-level group vs high-level group)	1.29	0.72–2.28	0.39
Anti-CD25c (low-level group vs high-level group)	1.11	0.62–1.97	0.73
Anti-FOXP3 (low-level group vs high-level group)	1.58	0.89–2.82	0.12

HR: hazard ratio; CI: confidence interval; yr: years; vs: versus.

## Discussion

The increased number of Treg cells in the circulation has been reported in several types of solid tumors, which is often associated with a poor outcome. The feasibility of detecting plasma anti-CD25 IgG was demonstrated in previous studies indicating the increased anti-CD25 IgG levels in NSCLC^25^ and esophageal cancer^23^. Subsequently, Liu and colleagues^22^ reported that patients with stage I breast cancer had a significantly higher anti-CD25 IgG level than healthy subjects, suggesting that the presence of a humoral immune response to Treg cells in cancer patients although the exact mechanism behind remains unclear. In the present study, we found that plasma levels of anti-CD25a and anti-FOXP3 IgG were significantly higher in patients with NSCLC than control subjects, and were gradually increased with NSCLC stages. It can be hypothesized that the number of CD25^+^FOXP3^+^ Treg cells may be positively correlated with NSCLC stages and increased release of CD25 and FOXP3 molecules may stimulate autoreactive B cells to secret more antibodies against these two molecules^28^. Unfortunately, the present study did not determine the proportion of circulating Treg cells, although such work could help to explain the role, if any, played by these natural autoantibodies. Further study will be performed to remedy this limitation.

In this study, we tested three individual peptide antigens derived from CD25 protein. Patients with NSCLC showed different pictures in plasma levels of IgG antibodies against these three CD25-derived antigens. In fact, each protein can carry many different epitopes that can be recognized by B cell receptors (BCRs), and trigger different immune responses^28^. It is worth noting that plasma anti-CD25b IgG levels were significantly decreased in an early stage of NSCLC as compared with control subjects (Table 4). This observation raises the possibility that natural anti-CD25 antibodies may be particularly important to maintain homeostasis of the immune system and decreased anti-CD25 IgG levels may undermine the function of immune surveillance in healthy individuals, leading to the development of cancer.

Our findings in this work appeared to be inconsistent with the results reported by previous studies demonstrating that patients with NSCLC had an increase in plasma anti-CD25b IgG levels^24,25^ and that plasma anti-FOXP3 IgG levels were found to be increased in the early stage of esophageal cancer^27^. This inconsistency may be attributable to the difference in tumor types, sample size, ELISA methods, small power or skewed sampling. The most possible reason might be the different experimental methods used between different studies. In this work, we applied a larger sample size with a good reproducible ELISA to detect plasma IgG levels, so that this work should be more conclusive and meaningful.

In summary, natural autoantibodies and Treg cells, as key components of the immune surveillance system, may play a crucial role in destruction of malignant cells formed in the body. A recent report indicated that anti-CD25 antibody could efficiently deplete intra-tumoral Treg cells through Fcγreceptor (FcγR) mediated mechanism in a murine model^29^. Natural anti-CD25 antibodies may play a similar role in developing anticancer activity in humans. They may inhibit the function of Treg cells through a variety of mechanisms such as neutralization and clearness of the excess of Treg cells via FcγR mediated phagocytosis. Reduced levels of natural anti-CD25 antibodies may lead to a high risk of cancer development.

## Methods

### Participants

Plasma samples were collected from 211patients with NSCLC who were admitted to the Department of Thoracic Surgery, China-Japan Union Hospital of Jilin University in the period between November 2012 and August 2016; 200 control subjects were simultaneously recruited from local communities. All the details regarding recruitments have been detailed in our previous report^30^. The demographic and clinical information is given in Supplementary Table S2. All patients underwent radiographic examination and histological confirmation; their plasma samples were obtained after diagnosis but prior to any anticancer treatment given. Only patients with adenocarcinoma and squamous cell carcinoma were included in this study. Patients with NSCLC were individually well matched for age, gender and smoking history with control subjects who underwent clinical interview and radiographic or imaging examination to exclude those had evidence of malignant tumors and autoimmune diseases that may affect the production of natural autoantibodies. To explore whether plasma IgG levels of CD25 and FOXP3 were altered in the early stage of NSCLC, these patients were divided into four subgroups based on the TNM (tumor, node and metastasis) staging system: group I for stage T_1_N_0_M_0_, group II for stage T_1_N_1_M_0_ + T_2_N_0_M_0_, group III for stage T_2_N_1_M_0_ + T_3_N_0_M_0_ and group IV for stages 3 and 4. Follow-up information of patients with NSCLC were obtained from the Large-scale Data Analysis Center of Cancer Precision Medicine-LinkDoc database^31^. All the participants were of Chinese Han origin and all provided written informed consent to participate in the study for the pathogenesis of lung cancer. This study was approved by the Ethics Committee of Second Hospital of Jilin University and conformed to the Declaration of Helsinki.

### Detection of plasma IgG levels

Four linear peptide antigens, including 3 derived from CD25 namely CD25a, CD25b and CD25c and 1 from FOXP3 (Supplementary Table S3), were designed using a computational epitope prediction software (http://www.iedb.org) based on the features of the target proteins such as hydrophilicity, flexibility, surface accessibility and antigenicity; they were then synthesized by solid-phase chemistry with a purity of >95%. The in-house ELISA was developed as described previously^30,32,33^. All assays were performed in duplicate with this in-house ELISA; the specific binding ratio (SBR) that represent plasma IgG levels for CD25 and FOXP3 was used to present experimental data and calculated as follows:

$${\rm{SBR}}=({{\rm{OD}}}_{{\rm{sample}}}-{{\rm{OD}}}_{{\rm{NC}}})/({{\rm{OD}}}_{{\rm{PC}}}-{{\rm{OD}}}_{{\rm{NC}}})$$

Quality control (QC) sample pooled from ~100 plasma samples from unrelated healthy individuals was used for analysis of the inter-assay deviation, and CV was used to represent the reproducibility of the in-house ELISA.

According to Manufacturer’s instruction, total IgG levels in plasma were measured using IgG (Total) Human Uncoated ELISA Kit with Plates (Cat. 88–50550, Thermo Scientific).

### Data analysis

Kolmogorov–Smirnov one-sample test was used to analyse a normal distribution of plasma IgG levels. Due to the skewed distribution of plasma IgG levels in the control group (Supplementary Table S4), Mann–Whitney *U* test was applied to examine the differences in plasma IgG levels between NSCLC patients and control subjects. ROC curve analysis was performed to work out the AUC with 95% confidence interval (CI), and the sensitivity of ELISA antibody test against a specificity of ≥95%.

Patients who were successfully followed up were divided into two subgroups based on the medians of plasma IgG measurements: the low IgG level subgroup and the high IgG level subgroup. Kaplan-Meier survival analysis was performed to explore the difference in overall survival that was defined as the period between the date of first hospitalization and that of death or censoring between the low IgG level subgroup and the high IgG level subgroup. Univariate cox proportional hazard model was applied to determine prognostic factors.

### Data availability

The datasets generated and analysed in the present study are available from the corresponding author on reasonable request.

## Electronic supplementary material

Supplementary information
