# Supplementary material for: Alteration of circulating natural autoantibodies to CD25-derived peptide antigens and FOXP3 in non-small cell lung cancer
Source: Sci Rep. 2018 Jun 29;8:9847. doi: 10.1038/s41598-018-28277-1 (PMC6026197; doi:10.1038/s41598-018-28277-1)
Supplement: Supplementary file 1 — Supplementary information [file 41598_2018_28277_MOESM1_ESM.pdf]

Supplementary Information

Alteration of circulating natural autoantibodies to CD25-derived peptide antigens and FOXP3 in non-small cell lung cancer

Huan Zhao,[1] Xuan Zhang,[1*] Zhifeng Han,[2] Wenjing Xie,[1] Wei Yang,[1*] Jun Wei[3]

Supplementary Table S1.The inter-assay deviation estimated by analysis of QC samples (SBI)

TAAs	Mean±SD	N[a]	CV(%)[b]
CD25a	0.59±0.19	20	11.9%
CD25b	0.40±0.22	20	13.9%
CD25c	1.11±0.32	20	13.9%
FOXP3	0.56±0.24	20	12.3%

[a] The number of plates tested

[b] Coefficient of variation

Supplementary Table S2. Demographic and clinical information of NSCLC patients and control subjects

Characteristic	Patients	Controls
Age (years)	58.7±8.7	58.6±9.3
Gender		
Male	131 (62.1%)	103 (48.5%)
Female	80 (37.9%)	97 (51.5%)
Smoking history	106(50.2%)	91(45.5%)
Type of tumor		
Squamous cell carcinoma	87 (41.2%)	N/A
Adenocarcinma	124 (58.8%)	N/A

Supplementary Table S3. Information of peptide antigens derived from CD25 and Foxp3.

Antigens	Sequence(N→C)	NCBI accession	Position (aa)
CD25a	kpghcrepppweneateriyhfvvgqmvy	NP_000408	99-126
CD25b	iyhfvvgqmvyyqcvqgyralhrgpaesve	NP_000408	116-144
CD25c	khtsqfpgeekpqaspegrpesetsch	NP_000408	167-187
Foxp3	dwftrmfaffrnhpatwknairhnlslhkd	NP_001107849	331-358

Supplementary Table S4. Kolomogorov-Smirnov test for a normal distribution of plasma IgG levels.

Antibody	Skewness	Kurtosis	*P*
CD25a			
Patient	0.349	0.445	0.2
Control	0.554	0.136	0.014
CD25b			
Patient	1.786	4.049	<0.001
Control	1.59	3.443	<0.001
CD25c			
Patient	0.536	0.283	0.2
Control	0.644	1.161	0.026
Foxp3			
Patient	0.893	1.072	<0.001
Control	1.494	3.237	<0.001
Total IgG			
Patient	0.193	-0.326	0.2
Control	0.225	-0.414	0.028
